# Green Synthesised TiO_2_ Nanoparticles-Mediated *Terenna asiatica*: Evaluation of Their Role in Reducing Oxidative Stress, Inflammation and Human Breast Cancer Proliferation

**DOI:** 10.3390/molecules28135126

**Published:** 2023-06-29

**Authors:** Manjula M. Venkatappa, Chikkappa Udagani, Sujatha M. Hanume Gowda, Shivakumar Venkataramaiah, Ryan Casini, Ihab Mohamed Moussa, Rajeshwara Achur, Devaraja Sannaningaiah, Hosam O. Elansary

**Affiliations:** 1 Department of Biochemistry, Kuvempu University, Shankaraghatta, Shimoga 577451, India; manjusree199622@gmail.com (M.M.V.); sujatha.mh95@gmail.com (S.M.H.G.); 2 Department of Physics, University College of Science, Tumkur University, Tumkur 572103, India; drchikkappa19@gmail.com; 3 Centre for Bioscience and Innovation, Department of Studies and Research in Biochemistry, Tumkur University, Tumkur 572103, India; shivkumarv187@gmail.com; 4 School of Public Health, University of California, Berkeley, 2121 Berkeley Way, Berkeley, CA 94704, USA; ryan.casini@berkeley.edu; 5 Department of Botany and Microbiology, College of Science, King Saud University, P.O. Box 2455, Riyadh 11451, Saudi Arabia; imoussa1@ksu.edu.sa; 6 Plant Production Department, College of Food and Agriculture Sciences, King Saud University, P.O. Box 2455, Riyadh 11451, Saudi Arabia

**Keywords:** green synthesis, *Terenna asiatica*, TiO_2_ NPs, oxidative stress, inflammation, MCF-7 cells, anticancer activity

## Abstract

Oxidative stress and chronic inflammation interplay with the pathogenesis of cancer. Breast cancer in women is the burning issue of this century, despite chemotherapy and magnetic therapy. The management of secondary complications triggered by post-chemotherapy poses a great challenge. Thus, identifying target-specific drugs with anticancer potential without secondary complications is a challenging task for the scientific community. It is possible that green technology has been employed in a greater way in order to fabricate nanoparticles by amalgamating plants with medicinal potential with metal oxide nanoparticles that impart high therapeutic properties with the least toxicity. Thus, the present study describes the synthesis of Titanium dioxide nanoparticles (TiO_2_ NPs) using aqueous *Terenna asiatica* fruit extract, with its antioxidant, anti-inflammatory and anticancer properties. The characterisation of TiO_2_ NPs was carried out using a powdered X-ray diffractometer (XRD), Fourier transform infrared (FTIR), scanning electron microscopy (SEM), energy-dispersive X-ray diffraction (EDX), high-resolution transmission electron microscopy (HR-TEM), dynamic light scattering (DLS), and zeta-potential. TiO_2_ NPs showed their antioxidant property by scavenging 1,1-diphenyl-2-picrylhydrazyl (DPPH) free radicals in a dose-dependent manner with an IC_50_ value of 80.21 µg/µL. To ascertain the observed antioxidant potential of TiO_2_ NPs, red blood cells (RBC) were used as an in vitro model system. Interestingly, TiO_2_ NPs significantly ameliorated all the stress parameters, such as lipid peroxidation (LPO), protein carbonyl content (PCC), total thiol (TT), superoxide dismutase (SOD), and catalase (CAT) in sodium nitrite (NaNO_2_)-induced oxidative stress, in RBC. Furthermore, TiO_2_ NPs inhibited RBC membrane lysis and the denaturation of both egg and bovine serum albumin, significantly in a dose-dependent manner, suggesting its anti-inflammatory property. Interestingly, TiO_2_ NPs were found to kill the MCF-7 cells as a significant decrease in cell viability of the MCF-7 cell lines was observed. The percentage of growth inhibition of the MCF-7 cells was compared to that of untreated cells at various doses (12.5, 25, 50, 100, and 200 µg/mL). The IC_50_ value of TiO_2_ NPs was found to be (120 µg/mL). Furthermore, the Annexin V/PI staining test was carried out to confirm apoptosis. The assay indicated apoptosis in cancer cells after 24 h of exposure to TiO_2_ NPs (120 µg/mL). The untreated cells showed no significant apoptosis in comparison with the standard drug doxorubicin. In conclusion, TiO_2_ NPs potentially ameliorate NaNO_2_-induced oxidative stress in RBC, inflammation and MCF-7 cells proliferation.

## 1. Introduction

During oxidative stress, there is an imbalance between the formation of free radicals and the endogenous antioxidant system. Free radicals, at optimum, are levels involved in several cellular activities (cell proliferation, differentiation and apoptosis). Meanwhile, at higher concentrations they have a deleterious effect on tissue [1,2]. The mitochondrial respiratory chain is the key contributor to ROS, such as superoxide anion (O_2_), hydrogen peroxide (H_2_O_2_), hydroxyl radical (OH^•^), and organic peroxides [3,4]. Free radicals are the products of molecular oxygen reduction; the transfer of electrons to (O_2_) takes place in the mitochondrial membrane during electron transport chain [5,6]. When cells lack oxygen, the mitochondrial respiratory chain produces nitric oxide (NO) and reactive nitrogen species (RNS) [7]. RNS may further produce reactive oxygen species, such as aldehydes-malondialdehyde (MDA) and 4- hydroxynonenal (4-HNE), by stimulating excessive lipid peroxidation [8]. Proteins, lipids and DNA are the main targets for oxidative attack; hence, their modification leads to mutagenesis followed by cancer [9]. In addition, chronic inflammation caused by chemical, biological and physical factors are, in turn, accompanied by an augmented risk of several human cancers [10]. The epidemiological and experimental data revealed the interplay between chronic inflammation and various forms of cancers [11,12]. Breast cancer (BC) is more prevalent in women in the population, and leads to a high rate of mortality and morbidity worldwide [13]. The highest incidences of breast cancer were reported in Eastern Africa, Western Europe, and most developing regions. Perhaps the lowest incidences were reported in African countries [14,15]. Despite the advancement in the diagnostic procedures and mammographic screening that help reduce the cases of BC in developed countries, there perhaps remains a social stigma in underdeveloped countries [16]. Chronic inflammation plays a critical role in the cellular modification that elevates high levels of ROS (reactive oxygen species) generation and cell proliferation [17]. Cytokine-induced oxidative stress elicits breast carcinogenesis by causing genomic instability and malignant transformation [18]. Most importantly, the up-regulation of numerous pro-inflammatory proteins, such as COX-2, IL1-b, IL-8 and TNF-α, is one of the key factors for the pathogenesis of breast cancer [19]. Researchers also documented that high levels of COX-2 promote the generation of ROS and oestrogen could elicit breast carcinoma [20]. Currently, this cancer is managed by chemo-, radio-, hormone and target therapies and surgery at the benign stage [21]. However, these therapeutic options exert life-threatening side effects, thus herbal medicine and their isolated compounds are gaining much attention [21]. To mention a few of them, essential oils from *Syzygium aromaticum* and eugenol from Indian spices exhibit antioxidant and anticancer activity [22,23]. Over the last decades, nanomedicine-based cancer therapy has been considered as the most effective option due to its few side effects and high therapeutic index [24]. Despite several nanoparticles having been fabricated by encapsulating various synthetic and natural compounds, chitosan nanoparticles are the most sought-after natural biomaterial thus far. Several researchers documented the utility of chitosan nanoparticles in cancer treatment and drug delivery [25]. Metal theranostics appear to be a promising diagnostic and therapeutic tool for early cancer detection, treatment and targeted drug delivery [26]. For instance, organic (liposomes, polymeric micelles, dendrimers and nanocantilevers) and inorganic nanotools (silica, carbon, graphene, quantum dots and noble metal nanoparticles) have built a tendency of attraction in the scientific community due to their immense biological and medicinal utilities [27]. Titanium dioxide nanoparticles (TiO_2_ NPs) have garnered attention for their many pharmacological applications, such as in antibacterial, antifungal, anti-inflammatory, antiviral, anticancer and antidiabetic activities [28,29]. Even so, metal nanoparticles have been fabricated by employing several methods, such as UV radiation, laser ablation, lithography, aerosol technologies and photochemical reduction [30,31]. The scientific community have focused on the green synthesis of nanoparticles, using bacteria, yeast, fungi, and plants, due to them being cost effective and environmentally benign [27,32,33,34]. Among several natural resources, plants have been extensively used in green technology due to their stored phytochemicals, such as alkaloids, terpenes, saponins, phenols, alcohols and proteins, which can act as a reducing and capping agents. Most importantly, metal oxide nanoparticles synthesised by other methods were found to be toxic in nature, while metal oxide nanoparticles synthesised using green routes exhibit high therapeutic potential without toxicity. Therefore, the current study aims to fabricate TiO_2_ NPs via the green route method using *Terena assiatica* plant fruit extract and its ameliorating effect on oxidative stress, inflammation and human breast cancer (MCF-7).

## 2. Results

### 2.1. Synthesis and Characterisation of the TiO_2_ NPs

**Figure 1:**
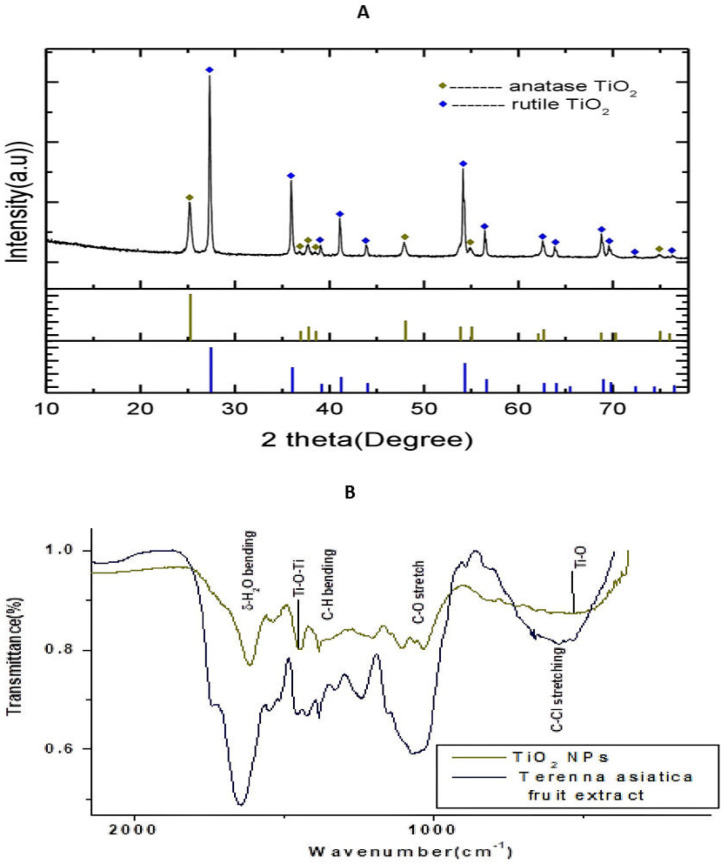
(**A**) Powder X-ray diffractometer pattern of TiO_2_ NPs. The high density peak at 2θ = 27.35° with interplanar spacing (d-spacing) 3.22 Å is due to (1 0 1) reflection of cubic TiO_2_. (**B**) Fourier transform infrared spectroscopy (FTIR) spectrum of TiO_2_ NPs and *Terenna asiatica* fruit extract showing stretching band.

The Powder X-ray Diffraction (PXRD) pattern of the NPs is depicted in the Figure 1A. The XRD peaks were well resolved and appeared sharp. The sharp peaks illustrated the high crystallinity of the NPs. The rutile peaks were observed at 2θ= 27.34°, 35.97°, 39.09°, 41.11°, 43.94°, 54.16°, 56.46°, 62.66°, 63.85°, 68.87°, 69.63°, 72.24° and 76.39°. These peaks were indexed for the reflection planes (1 1 0), (1 0 1), (2 0 0), (1 1 1), (2 1 0), (2 1 1), (2 2 0), (0 0 2), (3 1 0), (3 0 1), (1 1 2), (3 1 1), (3 2 0) and (2 0 2) of the tetragonal rutile TiO_2_ NPs (space group P4_2/mm_) according to the JCPDS card:21-1276. The XRD peaks, found at 2θ= 25.23°, 36.86°, 37.74°, 38.45°, 47.9°, 54.93° and 74.94°, were indexed for the reflection planes (1 0 1), (1 0 3), (0 0 4), (1 1 2), (2 0 0), (2 1 1) and (2 1 5) of the tetragonal anatase TiO_2_ NPs (space group l4_1/amd_) using the JCPDS card: 21-1272. The proportions of the anatase and rutile phases of TiO_2_ NPs were estimated using the formula:

(1) $$W_{A}=\frac{0.886I_{A}}{0.886I_{A}+I_{R}}\;W_{R}=\frac{I_{R}}{0.886I_{A}+I_{R}}$$

here, $W_{A}$ and $W_{R}$ denote the mass fraction of anatase TiO_2_ NPs and rutile TiO_2_ NPs, respectively, $I_{A}$ denotes the integral intensity of the major diffraction peak (1 0 1) of anatase TiO_2_ NPs and $I_{R}$ is the integral intensity of the major diffraction peak (1 1 0) of rutile TiO_2_ NPs. For the estimation of the integral intensity of the diffraction peaks, the software FityK was employed with baseline correction. The integral intensity of the diffraction peak due to (1 0 1) of anatase TiO_2_ NPs and the integral intensity of the diffraction peak due to (1 1 0) of rutile TiO_2_ NPs were, respectively, 42.28% ($I_{A}$) and 100% ($I_{R}$). Hence, the proportions of anatase TiO_2_ NPs and rutile TiO_2_ NPs were estimated to be 27.25% and 72.75%, respectively. Rutile TiO_2_ NPs had a higher stability than the anatase TiO_2_ NPs. The significant portion of rutile TiO_2_ NPs provided stability to the synthesised TiO_2_ NPs.

The Fourier transform infrared spectroscopy (FTIR) spectrum of the green synthesised TiO_2_ NPs depicts, and the *Terenna asiatica* fruit extract alone showed, the notable absorbance peaks at 3418 cm^−1^, 2923 cm^−1^, 2854 cm^−1^, 1610 cm^−1^, 1444 cm^−1^, 1380 cm^−1^, 1106 cm^−1^ and 540 cm^−1^ (Figure 1B). The deep absorption peak centred at 3418 cm^−1^ resulted from O-H stretch. The weaker peaks, found at 2923 cm^−1^ and 2854 cm^−1^, correspond to the C-H stretch, which confirmed the reduction in organic compounds. The peak centred at 1610 cm^−1^ is a characteristic of the δ-H_2_O bending. The absorbance peak found at 1444 cm^−^^1^ could be attributed to the stretching vibrations of Ti-O-Ti. The sharp peaks observed at 1380 cm^−1^ and 1106 ${cm}^{-1}$ were due to the presence of plant biocompounds in the synthesised TiO_2_ NPs and corresponded to the C-H bending and C-O stretch, respectively. The characteristic vibrational stretching mode of Ti-O was assigned to the broad band centred at 540 cm^−1^.

**Figure 2:**
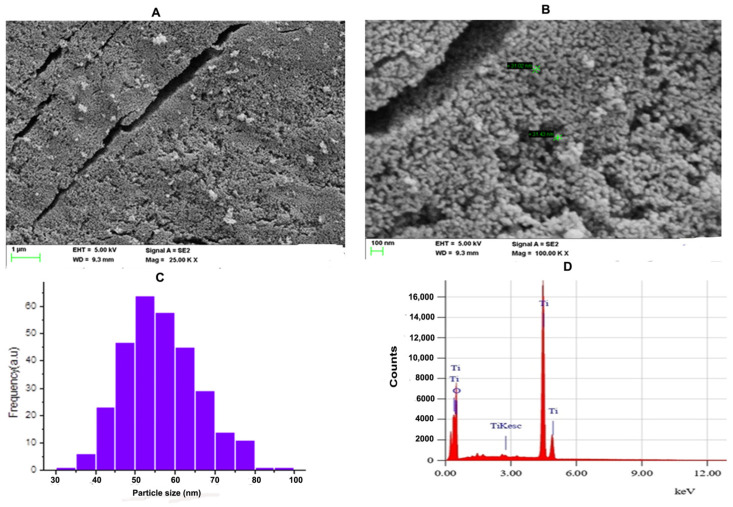
(**A**) FE-SEM microgram of TiO_2_ NPs; The SEM micrograph of TiO_2_ NPs was depicted with scale bar 1 μm. (**B**) The SEM micrograph of TiO_2_ NPs was depicted with scale bar 100 nm. (**C**) Histogram analysis for particle size calculation. (**D**) Energy-dispersive X-ray diffraction (EDX) reveals high elemental composition with Ti and O, showing high purity with mass percentage (%) of 40.07 and 50.39, respectively, and 4.253 and 0.625 are the keV of TiO_2_ NPs, respectively, confirming the formation of TiO_2_ NPs.

SEM is an effective technique for studying the surface morphology and nanocrystalline nature of a nanomaterial. Figure 2A shows the SEM micrograph of the TiO_2_ NPs with an accelerating voltage of 5 kV and magnification of 25.00 k at scale bar 1 μm. Figure 2B shows the SEM micrograph of the TiO_2_ NPs with an accelerating voltage of 5 kV and magnification of 100.00 k at scale bar 100 nm. The SEM micrographs showed that the particles have spherical morphology and the particles were distributed uniformly over the space without agglomeration. ImageJ was employed to know the size distribution of TiO_2_ NPs. Figure 2C indicates the histogram of particle size distribution TiO_2_ NPs. The size of nanoparticles ranges between 32.94 nm and 88.02 nm with a mean size of 56.54 nm. The elemental analysis of the TiO_2_ NPs was performed with energy-dispersive X-ray analysis (EDAX). Figure 2D represents the EDAX spectrum of the TiO_2_ NPs. The EDAX of the synthesised TiO_2_ NPs showed sharp peaks of oxygen (O) and titanium (Ti). Hence, no other impurity peaks were observed except O and Ti. The percentage compositions of oxygen and titanium in the synthesised TiO_2_ NPs was found to be 40.07% and 59.93%, respectively.

**Figure 3:**
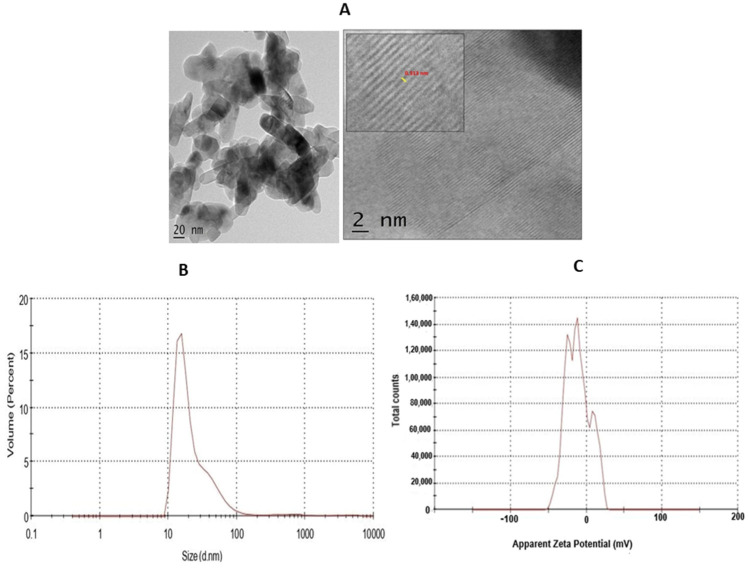
(**A**) HR-TEM of TiO_2_ NPs. The TEM micrograph of TiO_2_ NPs was depicted with scale bar 2 nm. (**B**) The dynamic light scattering (DLS) of TiO_2_ NPs depicted hydrodynamic size. (**C**) Zeta-potential analysis for particle charge calculation.

The high-resolution transmission electron microscopy (HRTEM) of synthesised TiO_2_ NPs, with a scale bar 2 nm, is represented in Figure 3A and shows a d-spacing of 0.313 nm. The dynamic light scattering (DLS) analysis is an effective technique to understand the hydrodynamic size of synthesised TiO_2_ NPs in solvent. The DLS measurement of the TiO_2_ NPs showed the concentration of TiO_2_ powder in water. The DLS analysis and zeta potential of TiO_2_ is depicted in Figure 3B. The DLS study showed the average hydrodynamic diameter of 125.9 nm with a polydispersity index value of 0.540. The average zeta potential was found to be −11.1 mV. The negative net zeta potential is attributed to the attachment of negative moieties on the surface of TiO_2_ NPs. The negative zeta potential result indicates the stability of the NPs (Figure 3C).

### 2.2. TiO_2_ NPs Ameliorates the Oxidative Stress Markers of NaNO_2_-Stress-Induced RBC Cell through its Antioxidant Potential

**Figure 4:**
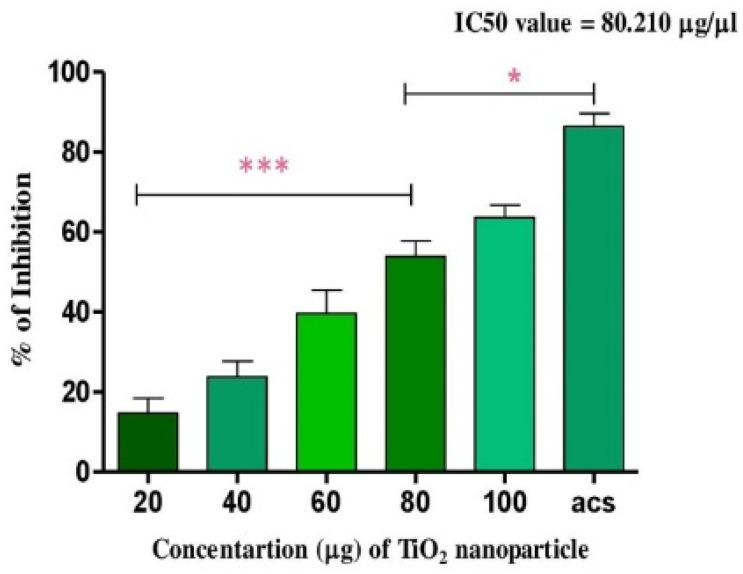
Antioxidant activity of TiO_2_ NPs: the DPPH free radical scavenging potential of TiO_2_ NPs examined by measuring its antioxidant activity in comparison with ascorbic acid. Each value is given as a mean ± SD. * Significance at *p* ≤ 0.005 and *** at *p* ≤ 0.001.

**Figure 5:**
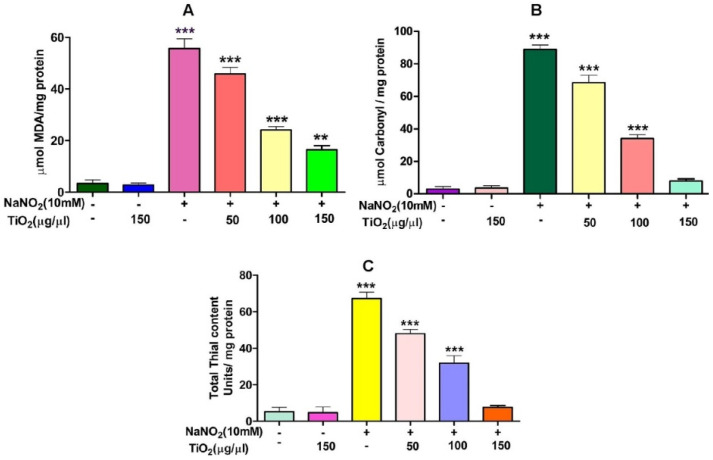
Role of TiO_2_ NPs on NaNO_2_-induced oxidative stress in red blood cells: (**A**) lipid peroxidation, (**B**) protein carbonyl content and (**C**) total thiol content. To identify the protective effect of TiO_2_ NPs against NaNO_2_-induced oxidative damage, red blood cells were pre-incubated for 10 min with various concentrations (50–150 µg/mL) of TiO_2_ NPs at 37 °C before treatment with NaNO_2_ (10 mM). The results are presented in average units/mg of protein and are expressed as mean ± SEM (n = 3). Significance at *p* ≤ 0.005, ** at *p* ≤ 0.001 and *** at *p* ≤ 0.0001.

**Figure 6:**
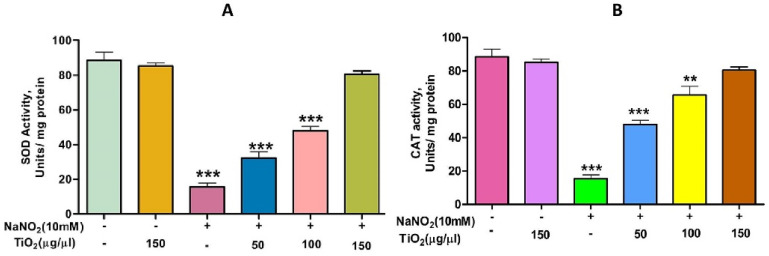
Role of TiO_2_ NPs on NaNO_2_-induced oxidative stress in red blood cells: (**A**) super-oxide dismutase (SOD) and (**B**) catalase (CAT). To examine the protective role of TiO_2_ NPs against NaNO_2_-induced oxidative damage, red blood cells were pre-incubated for 10 min with different concentrations (50–150 µg/mL) of TiO_2_ NPs at 37 °C before treatment with NaNO_2_ (10 mM). The results are presented in average units/mg of protein and are expressed as mean ± SEM (*n* = 3). Significance at *p* ≤ 0.005, ** at *p* ≤ 0.001 and *** at *p* ≤ 0.0001.

TiO_2_ NPs scavenged the Di-Phenyl-2-Picryl-Hdrazyl (DPPH) free radical in vitro in a concentration-dependent manner, suggesting its antioxidant property. The antioxidant potential of TiO_2_ NPs was found to be 73.92% in comparison with the positive control ascorbic acid (88%) with an IC_50_ value of 42.71 µg (Figure 4). The antioxidant potential of TiO_2_ NPs was further evaluated in NaNO_2_-induced oxidative stress RBC model. The levels of LPO, PCC and TT in the treated RBC samples were investigated as the key oxidative stress biomarkers. By comparing the level of malondialdehyde, the amount of lipid peroxidation (LPO) was identified. RBCs treated with NaNO_2_ (10 mmol/L) had a significantly higher MDA concentration, while TiO_2_ NPs (0–150 µg) dose-dependently decreased the MDA level in RBCs. It is possible that the MDA level did not change in the TiO_2_ NPs alone treated group (Figure 5A). The impact of TiO_2_ NPs on protein oxidation in RBCs lysate was assessed; RBCs lysate treated with NaNO_2_ showed a significant increase in protein carbonyl content (PCC) compared to the control. In contrast, the level of PCC was intensely recovered in the TiO_2_ NPs (0–150 µg) that had been pre-incubated with RBC as compared to the control sample (Figure 5B). Additionally, the level of total thiol content was also higher in RBC that was exposed to NaNO_2_. In comparison to the control, the total thiol content of TiO_2_ NPs (0–150 µg) that was pre-treated with RBC was found to be reduced (Figure 5C). Interestingly, when TiO_2_ NPs (0–150 µg) were treated with RBC alone, none of the three metrics changed from the control RBC. Furthermore, in NaNO_2_-treated RBCs compared to the control, the activity of antioxidant enzymes like SOD and CAT were dramatically decreased (Figure 6A,B), whileTiO_2_ NPs (0–150 µg) significantly normalised the activity of SOD and CAT. However, the level of SOD and CAT did not change in the TiO_2_ NPs alone treated group.

### 2.3. Anti-Inflammatory Activity of TiO_2_ NPs

**Figure 7:**
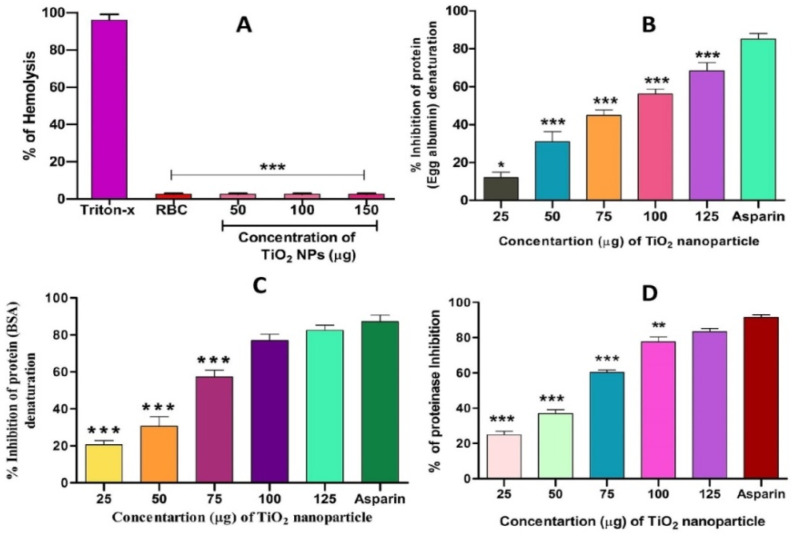
Effect of TiO_2_ NPs on protein denaturation: (**A**) % of hemolysis, (**B**) % inhibition of egg albumin denaturation, (**C**) % inhibition of BSA denaturation and (**D**) % of proteinase inhibition. The results are presented in average units/mg of protein and are expressed as mean ± SEM (*n* = 3). * Significance at *p* ≤ 0.005, ** at *p* ≤ 0.001 and *** at *p* ≤ 0.0001.

Interestingly, TiO_2_ NPs exhibited anti-haemolytic activity by inhibiting RBC membrane lysis compared to the positive control aspirin (Figure 7A). The anti-inflammatory activity of TiO_2_ NPs was further confirmed by the denaturation of egg albumin and bovine serum albumin (Figure 7B,C). Interestingly, TiO_2_ NPs inhibited the denaturation of both egg and bovine serum albumin significantly in a dose-dependent manner. The maximum percentage of inhibition of protein denaturation by TiO_2_ NPs was found to be 84.64% and 76.95% for egg albumin and bovine serum albumin, respectively, which was compared with standard drug aspirin (90% inhibition). During inflammation, serine protease is the key enzyme released from neutrophils and it plays an important role in tissue damage during the inflammatory response. Thus, a trypsin inhibition assay was carried out. Surprisingly, TiO_2_ NPs inhibited trypsin significantly in a dose-dependent manner compared to the positive control aspirin (Figure 7D). The observed trypsin inhibition was found to be 80%, which well agreed with the positive control aspirin (94%).

### 2.4. Anticancer Activity

#### TiO_2_ NPS Alters the Morphology of MCF-7 Cells

**Figure 8:**
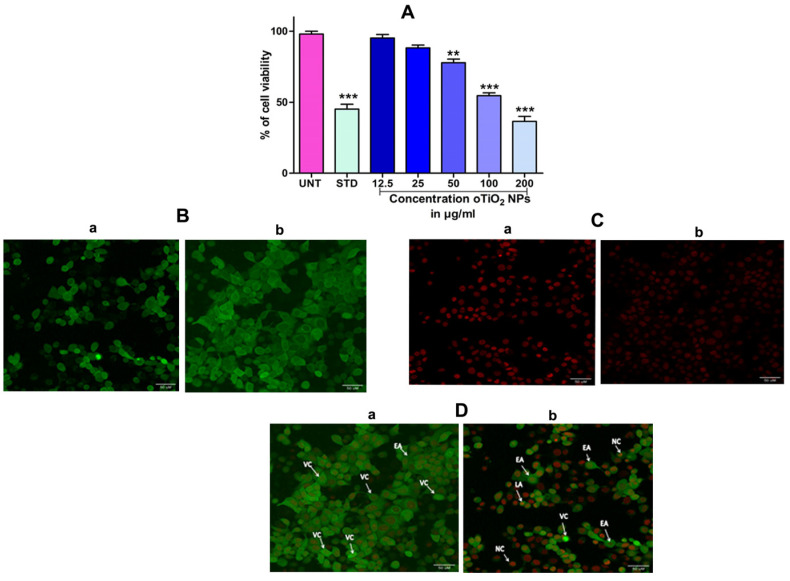
(**A**) Percentage of cell viability of TiO_2_ NPs against the human breast cancer (MCF-7) cell lines after the treatment period of 24 h. (**B**) Acridine orange (AO): (**B**(**a**)) acridine orange staining of untreated cell, (**B**(**b**)) acridine orange staining of TiO_2_ NPs-treated cell. (**C**) Ethidium bromide (EB): (**C**(**a**)) ethidium bromide staining of untreated cell, (**C**(**b**)) ethidium bromide staining of TiO_2_ NPs-treated cell. (**D**) Dual-staining cells: (**D**(**a**)) staining of untreated cell, (**D**(**b**)) staining of TiO_2_ NPs-treated cell: dual staining of TiO_2_ NPs by fluorescence microscopy with an IC_50_ concentration representing the changes in nuclear morphology of cells. AO represents viable cells (green) and EtBr represents dead cells (red colour). The images were captured at 40× magnification. The results are expressed as mean ± SEM (n = 3). Significance at *p* ≤ 0.005, ** at *p* ≤ 0.001 and *** at *p* ≤ 0.0001.

**Figure 9:**
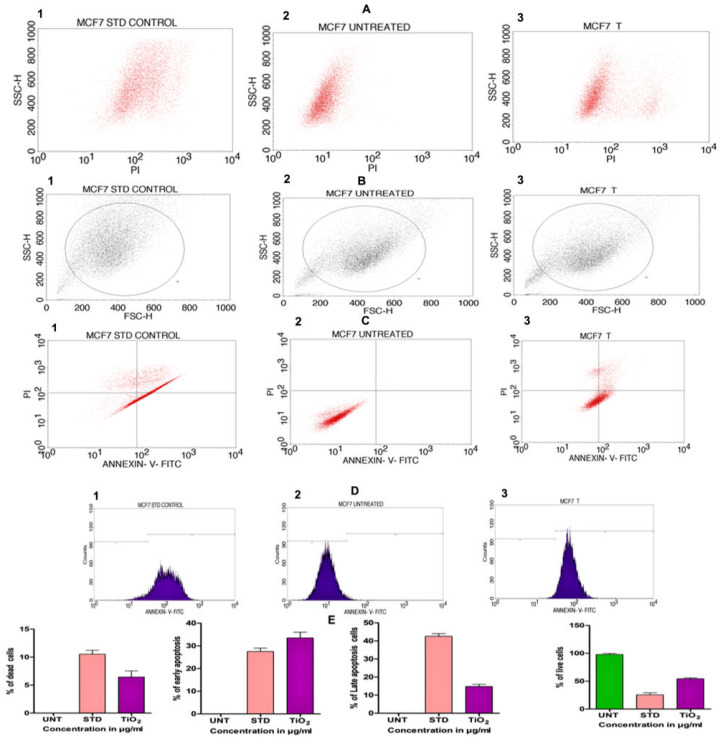
(**A**) Evaluation of cell death by propidium iodide (PI)/side scatter (SSC-H): (**A1**) cells treated with positive control doxorubicin (standard), (**A2**) untreated cells, (**A3**) cells treated with TiO_2_ NPs. (**B**) Evaluation of cell death by forward scatter (FSC-H)/side scatter (SSC-H): (**B1**) cells treated with positive control doxorubicin (standard), (**B2**) untreated cells, (**B3**) cells treated with TiO_2_ NPs. (**C**) Quadrangular plots representing the Annexin-V/PI expression in MCF-7 cells upon culturing in the presence and absence of TiO_2_ NPs: (**C1**) cells treated with positive control doxorubicin (standard), (**C2**) untreated cells, (**C3**) cells treated with TiO_2_ NPs. (**D1**–**D3**) Micrograph represents the percentage of cells (**E**) % of live, apoptotic and necrotic cells in comparison to standard drug (Doxorubicin) and TiO_2_ NPs.

In order to assess the cytotoxicity of the TiO_2_ NPs in MCF-7 cells, a 3-(4,5-Dimethylthiazol-2-Yl)-2,5-diphenyl-tetrazolium bromide (MTT) cell viability experiment was performed. The percentage of growth inhibition of the MCF-7 cells was compared to that of untreated cells at various doses of TiO_2_ NPs (12.5, 25, 50, 100, and 200 µg/mL) and this showed a significant decrease in the cell viability of the MCF-7. The IC_50_ value for cells treated with TiO_2_ NPs was 120 µg/mL at this concentration and showed 50% growth inhibition. Figure 8A depicts the concentration-dependent growth inhibition of MCF-7 cells. Furthermore, the morphological alterations of MCF-7 cells treated with TiO_2_ NPs were examined using the Acridine orange(AO)/Ethidium bromide (EtBr) fluorescent microscopic staining test (Figure 8B,C). MCF-7 cells that were untreated with TiO_2_ NPs did not take up the AO stain, suggesting they were alive with normal morphology, and remained green in colour (Figure 8D(a)). On the other hand, TiO_2_ NPs-treated MCF-7 cells, after 24 h at a concentration of 120 µg/mL, showed changes in the nucleus morphology due to apoptosis and took up a red colour upon EtBr interaction (Figure 8D(b)). The apoptosis caused by TiO_2_ NPs was further authenticated using Annexin V/PI using a flow cytometer. TiO_2_ NPs, upon exposure for about 24 h with MCF-7 cells, showed significant apoptosis with an IC_50_ value of 120 µg/mL that was compared to the positive control doxorubicin. Figure 9A shows the evaluation of cell death by propidium iodide (PI)/ side scatter (SSC-H), Figure 9B depicts the evaluation of cell death by forward scatter (FSC-H)/side scatter (SSC-H) and the Annexin-V/PI expression in MCF-7 cells upon culturing in the presence of TiO_2_ NPs (Figure 9C). The flow cytometry of TiO_2_ NPs is represented in the micrograph (Figure 9D). In each case, the early and late apoptotic cell populations were 26.67% and 41.37%, respectively, for doxorubicin, while TiO_2_ NPs-treated MCF-7 cells showed early and late apoptosis with death rates of 13.63% and 30.17%, respectively. In contrast, the untreated cells showed no significant apoptosis (Figure 9E).

## 3. Discussion

Recently, green nanotechnology appears to be most promising for its high efficacy, eco-friendly and low cost properties without toxic effect [35,36]. *Terenna asiatica* is a medicinal herb with a wide array of therapeutic potential and it has been used in folk medicine since ancient times [37]. The therapeutic utility of *Terenna asiatica* could be the effect of robust phytochemicals, namely alkaloids, tanins, phenolics and flavonoids. These secondary metabolites play key roles as reducing, oxidising, chelating and stabilizing agents [38,39,40]. Even so, the efficient biogenic synthesis of TiO_2_ NPs using medicinal plants with antibacterial, antimicrobial and anticancer properties has been documented [41,42]. The therapeutic effect of green synthesised TiO_2_ NPs using *Terenna asiatica* fruit extract is least studied. Therefore, the current study showcases green fabricated TiO_2_ NPs using *Terenna asiatica* fruit extract that exhibits potential antioxidant, anti-inflammatory and anticancer properties. TiO_2_ NPs were characterised for size distribution, morphology, surface charge and surface chemistry [43,44,45] using various techniques. The powder X-ray diffraction (PXRD) technique is the most revered technique for the structure and phase confirmation of nanomaterial. The TiO_2_ NPs have both anatase and rutile phases. However, the rutile phase constitutes the major portion. There are no other impurity peaks in the observed XRD pattern. The absence of impurity peaks showed the high purity of the NPs [46,47,48,49,50]. FTIR analysis was carried out using a range between 500 cm^−1^ and 4000 cm^−1^. The FTIR spectrum of the green synthesised NPs depicts the notable absorbance peaks [51,52,53]. The SEM micrographs showed that the particles have spherical morphology and the size of the nanoparticles is 56.54 nm [54]. Furthermore, a conformation of synthesised nanoparticles was carried out by using HR-TEM, which showed d-spacing of 0.313 nm [55]. The nature of the hydrodynamic size was analysed by DLS, and it showed 125.9 nm [56], and the zeta potential of TiO_2._ NPs was found to be −11.1 mV [57]. During oxidative stress, ROS/RNS cause lipid peroxidation, protein carbonylation and thiolation that modulate gene expression by damaging genetic material, leading to the inflammation, tissue damage and cell apoptosis that are considered as the hall marks of cancer [58]. TiO_2_ NPs exhibited an antioxidant property by scavenging DPPH in vitro. These data were also authenticated using an in vivo RBC model, TiO_2_ NPs efficiently normalised the altered level of stress parameters such as lipid peroxidation (LPO), protein carbonyl content (PCC), total thial (TT) and antioxidant enzymes (catalase and super oxide dismutase). Previous studies reported that the metal oxide nanoparticles, such as Mgo, protect RBC from oxidative damage [37].

Researchers proved that, at higher levels, ROS/RNS tend to initiate the inflammatory process by producing more of the pro-inflammatory cytokines, nuclear protein kappa B/active protein −1 and tumour necrosis factor alpha responsible for numerous chronic diseases including cancer [59]. In addition, human red blood cell (HRBC) membrane stabilisation is one of the most important techniques adopted to examine anti-inflammatory activity [60]. The RBC membrane is similar to the lysosomal membrane. The stabilisation of the lysosomal membrane plays a key role in the inflammatory response by preventing the release of neutrophils in specific bacterial proteins and proteases that damage upon extracellular discharge, resulting in chronic and acute inflammation [61]. Thus, the inhibition of the HRBC membrane helps to maintain its integrity. Interestingly, TiO_2_ NPs exhibited anti-haemolytic activity by inhibiting RBC membrane lysis compared to the positive control aspirin. Protein denaturation is implicated in arthritic responses and tissue damage progression during inflammation [62]. Our results reveal that TiO_2_ NPs were effective in inhibiting thermally induced haemolysis and also in the denaturation of protein (egg and bovine serum albumin) in all tested doses, showing the capability of controlling the protein denaturation involved in the inflammatory process. During inflammation, serine protease is the key enzyme released from neutrophils and it plays an important role in tissue damage during the inflammatory response. TiO_2_ NPs inhibited trypsin compared to the positive control aspirin, supporting its observed anti-inflammatory potential. Several metal and metal oxide NPs have been reported to be endowed with anti-inflammatory properties like silver [63], gold [64], selenium [65], copper [66], nickel [67], zinc oxide [68], zinc peroxide [69], cerium oxide [70], iron oxide [71] and titanium dioxide [72].

During oxidative stress, the key transcription factors, namely NF-κB, AP-1, p53, HIF-1α, PPAR-γ, β-catenin/Wnt, and Nrf2, are upregulated/activated. The upregulation of said transcription factors results in the expression of more than 500 genes, including growth factors, inflammatory cytokines, chemokines, cell cycle regulatory molecules and anti-inflammatory molecules. Overall, research validates that oxidative stress, chronic inflammation and various types of cancer are closely related. Chemotherapeutic drugs (doxorubicin, epirubicin, paclitaxel, doxetaxel, vinorelbine, methotrexate, gemcitabine, cisplastin and chromboplastin) are known to cause DNA damage, to impair cognitive function, to cause anaemia, pulmonary toxicities, colitis, diarrhoea, cardio toxicity, gastro intestinal tract toxicity, weight gain, jaundice, diarrhoea and loss of bone density [73]. Thus, green nanotechnology opens a new avenue and offers better treatment options in comparison to chemically synthesised drugs. TiO_2_ NPs exhibited anticancer activity, as confirmed by cytotoxicity, morphological studies by fluorescence and cell apoptosis studies. TiO_2_ NPs caused higher cytotoxic effect to MCF-7 cell lines by inhibiting their cellular growth. In addition, they caused apoptosis, a programmed cell death marked by morphological changes such as nuclear fragmentation, chromatin condensation, blebbing of the plasma membrane and the presence of apoptotic bodies. Therefore, our findings indicate that the TiO_2_ NPs synthesised by green technology appear to be promising for the better management of oxidative-stress-induced cancer as well. Green synthesised nanoparticles, such as gold, silver, iron oxide, zinc oxide and titanium dioxide, showed anticancer properties [74,75,76,77,78].

## 4. Materials and Methods

### 4.1. Chemicals and Reagents

The chemicals and reagents used were: titanium hexa nitrite, methanol, DPPH (1,1-diphenyl-2-picrylhydrazyl), NaNO_2_ (sodium nitrate), EDTA (ethylene diamine tetra acetic acid), TEMED (tetra methyl ethylene diamine), DNPH (2,4-dinitro phenyl hydrazine), TCA (Tri chloro acitic acid), DTNB (5,5′-dithiobis-(2-nitrobenzoic acid)-Elman’s reagent, SDS (sodium dodacyle sulfate), acetic acid, thiobarbituric acid, H_2_O_2_ (hydrogen peroxide), DNS (3,5 dinitro salicylic acid), NaOH (sodium hydroxide), sodium potassium tartarate, starch, PBS (phosphate buffer saline), BSA (bovine serum albumin), egg albumin and trypsin.

### 4.2. Preparation of Fruit Extract

Fruits of the *Terenna asiatica* plant were collected from Siddara Betta, Tumkur District, Karnataka State, India. Species name of the plant, its genera and class were identified and authenticated by the Department of Botany, Tumkur University, Tumkur. The specimens were kept in the department. They were carefully cleaned and allowed to air dry for 48 h at room temperature. Using a pestle and mortar, the fruits were mechanically homogenised and powdered. A known quantity of deionised water was added along with the weighed powder (4.8 g). The suspension was blended and stirred for two hours at 40 °C. By using Whatman no-1filter paper, the cooled mixture was filtered.

### 4.3. Synthesis of TiO_2_ NPs by Green Route Method

Aqueous *Terenna asiatica* fruit extract was used to synthesis TiO_2_ nanoparticles. To synthesize TiO_2_ nanoparticles, we dissolved 1.5 N of titanium tetra isopropoxide in 100 mL of distilled water. Drop by drop with constant stirring, extract was added until pH of solution reached room temperature; the mixture was constantly stirred for 3 h. The obtained nanoparticles were separated using filter paper and the materials were continuously rinsed with distilled water to remove the by-product. The obtained wet nanoparticles were dried at 800 °C overnight. To obtain rutile phase, particles were finally calcined at 600 °C for 3 h [79].

### 4.4. Characterisation of TiO_2_ NPs

The UV-Visible absorption spectrum was recorded using a UV-Visible spectrophotometer (UV) (UV-1800; Shimadzu, Tokyo, Japan). A Fourier transform infrared (FTIR) spectrometer (Perkin Elmer, Spectrum 400, Waltham, MA, USA) was used to analyse the chemical bonding of the synthesised nanoparticles and an energy-dispersive X-ray diffraction (EDX) (JCM-6000PLUS, New Delhi, India) system was used to determine the elemental composition of the TiO_2_ nanoparticles. Characteristic peaks were observed by X-ray diffraction detector (XRD) (Bruker D8 Advance XRD diffractometer, Bruker, Karlsruhe, Germany), the size and the morphology of the TiO_2_ nanoparticles were determined by using scanning electron microscope (SEM) (JCM-6000PLUS, New Delhi, India), d-spacing between the particles was carried out by using high-resolution transmission electron microscopy (HR-TEM) (Jeol/JEM 2100, JEOL Inc., Peabody, MA, USA) and dynamic light scattering (DLS) and zeta potential were carried out by using Malvern Zetasizer (Malvern, Worcestershire, UK, WR14 1XZ).

### 4.5. In Vitro Antioxidant Activity

#### DPPH Assay

The fraction’s free radical scavenging activity was measured in vitro using the 1,1-diphenyl-2-picrylhydrazyl (DPPH) assay. A 10 mM solution of DPPH in 50% methanol was prepared, and TiO_2_ NPs (0–100 µg) were added and made up to 2.5 mL using methanol before being thoroughly mixed. Then, 140 µL of a 10 mM DPPH reagent was added. Using a UV-Vis spectrophotometer, a reading of 517 nm was obtained after the mixture had been thoroughly mixed and incubated at room temperature for 30 min [80]. Ascorbic acid was used as a standard drug control. The following formula was used to determine the percentage of DPPH inhibition:

% DPPH inhibition = Ab_c_ − Ab_s_ × 100/Ab_c_ (2)

where Ab_c_—absorbance of control, Ab_s_—absorbance of sample.

### 4.6. Lipid Peroxidation (LPO) Assay

Red blood cells were drawn from a healthy human being and made into 2% hematocrit by using phosphate buffer with 7.4 pH. Reaction mixture consisted of 2 mg of protein (200 µL of RBC lysate) and different concentrations of TiO_2_ NPs (0–100 µg). Lipid peroxidation was induced by 10 mM NaNO_2_ and it was incubated for 30 min at room temperature. Then, 1.5 mL acetic acid (pH 3.5), 0.2 mL of 8% SDS and 1.5 mL TBA (0.8%) were added to the reaction mixture and it was incubated for 45 min at 45–60 °C after butanol and pyridine were added in the ratio 2:1. The reaction was mixed thoroughly and centrifuged at 3000 rpm for 15 min and we read the absorbance at 530 nm [81].

### 4.7. Estimation of Protein Carbonyl Content (PCC)

A previously described method of using DNPH was followed to measure the protein carbonyl content [82]. Briefly, 1 mL of RBC lysate (2 mg protein/mL) was treated with NaNO_2_ (10 mM) and TiO_2_ NPs (0–100 µg) and incubated for 30 min. Later, 0.5 mL of 10 mM DNPH in 2 N HCl was added, and the mixture was incubated for 1 h at room temperature while being shaken occasionally. By only adding 2 N HCl to the sample, the corresponding blank was performed. After incubation, the mixture was centrifuged after being precipitated with 20% TCA. The precipitate was washed twice with acetone before being dissolved in 1 mL of Tris buffer (20 mM pH 7.4 with 0.14 M NaCl, 2% SDS), and the supernatant’s absorbance was measured at 360 nm. We calculated and expressed how the absorbance varies in nmols of carbonyl groups/mg of protein.

### 4.8. Measurement of Total Thiols (TT)

The method described in [83] was used to assess the total thiols. To 1 mL of RBC lysate (2 mg protein/mL), NaNO_2_ (10 mM) and TiO_2_ NPs (0–100 µg) were added and incubated for 30 min. An amount of 0.375 mL of 0.2 M Tris-HCl buffer (pH 8.2) was vortexed and we incubated the mixture for 30 min with 10 mM dithiol-bis-nitro benzoic acid (DTNB) and 1.975 mL of methanol. The tubes were centrifuged at 5000 rpm for 10 min. The samples’ clear supernatants were collected and measured for photometric absorbance at 412 nm, with the thiol content expressed as n mol of DTNB oxidised/mg protein.

### 4.9. Superoxide Dismutase (SOD) Assay

The method of Sundaram et al. was used to measure the SOD enzyme activity [84]. In clean, dry test tubes, 2 mg of protein from the TiO_2_ NPs (0–100 µg) treated RBCs, with an agonist NaNO_2_ (10 mmol/L), was added with 1 mL of the reaction mixture, which was composed of phosphate buffer (16 mmol/L, pH 7.8) and TEMED-EDTA (8 mmol/L/0.08 mmol/L) mixture. At 406 nm, the decrease in absorbance was observed for 3 min. The results were expressed in terms of U/ mg of protein.

### 4.10. Catalase (CAT) Assay

Shangari’s method was employed to measure the CAT enzyme activity [85]. In clean, dry test tubes, 2 mg of protein from TiO_2_ NPs (0–100 µg) treated RBCs, with an agonist NaNO_2_ (10 mM), was added. The reaction mixture also contained sodium phosphate buffer (100 mM/L, pH 7.4) and H_2_O_2_ (8.8 mM/L). For 3 min, the absorbance was measured at 240 nm. The CAT activity was expressed as U/mg of protein.

### 4.11. In Vitro Anti-Inflammatory Activity

#### 4.11.1. Membrane Stabilisation Activity by Hemolytic Assay

As previously mentioned by [86], the direct hemolytic activity was measured. An amount of 9 mL of 10 mM PBS and approximately 1 mL of human red blood cells were added (pH 7.4). Then, 1 mL of these diluted blood samples were incubated for 1 h at 37 °C with various TiO_2_ NPs concentrations (0–150 µg). With the addition of 9 mL of ice-cooled 10 mM PBS, the haemolytic reaction was stopped (pH 7.4). The samples were centrifuged for 10 min at 37 °C at 1500 rpm. To assess the degree of hemolysis, the amount of released haemoglobin in the supernatant of the samples was calculated and compared at 540 nm. Tritonx-100 was used as a positive control and RBC alone was a positive control. The percentage of hemolysis was calculated by using following formula:

% inhibition of hemolysis = 1 − absorption of test sample/absorption of the control × 100 (3)

#### 4.11.2. Protein Denaturation Assay

The method described by [87] was followed for the protein denaturation assay. The reaction mixture contained TiO_2_ NPs (0–100 µg), 4.78 mL of phosphate buffered saline (PBS, pH 6.4) and 0.2 mL of 1% bovine albumin and egg albumin. The mixture was incubated at 37 °C for 15 min before being heated in a water bath at 70 °C for 5 min. A UV-Visible spectrometer was used to measure the turbidity at 660 nm after cooling. As a control, phosphate buffer saline and the drug control aspirin were used. The following formula was used to calculate the percentage of denaturation:

% of inhibition of denaturation = 1 − absorbance of test sample/absorbance of the control × 100 (4)

#### 4.11.3. Protease Inhibition Assay

The TiO_2_ NPs were tested for their ability to inhibit serine protease using the technique described in [88]. The reaction mixture contained 0.06 mg trypsin, 1 mL of 20 mM Tris-HCl buffer (pH 7.4) and TiO_2_ NPs (0–100 µg). The mixture was first incubated at 37 °C for 5 min, later 1 mL of 0.8% (*w*/*v*) casein was added and the process was repeated for 20 min. To stop the reaction, 2 mL of 70% per chloric acid was added after the incubation time. Then, the mixture was centrifuged at 3000 rpm for 10 min, the supernatant was collected and absorbance was measured at 210 nm using buffer as the reference. Phosphate buffer saline and the drug aspirin were used as the controls. The percentage of denaturation was calculated using the Formula (4).

### 4.12. Anti-Cancer Activity

#### 4.12.1. Cell Line Studies

The method described by [89] was followed for the cell line studies. MCF-7-human breast adenocarcinoma cell line was purchased from NCCS, Pune Culture Collection. MCF-7 cells were grown in DMEM-high glucose media (modified eagle medium from Dulbecco’s) with 10% FBS as a dietary supplement. All cell lines were preserved at 37 °C in humidified 5% CO_2_ and 95% atmospheric air. We incubated cells in a 6-well plate at a density of 0.5 × 106 cells/2 mL overnight at 37 °C in a CO_2_ incubator. Using 2 mL of culture medium, we treated the cells with the required concentration of the experimental compounds and the controls and then incubated the cells for 24 h. We provided a PBS wash to each well later. We incubated them at 37 °C for 3–4 min after removing the PBS and adding 200 μL of trypsin-EDTA solution.

#### 4.12.2. MTT Assay

The method described by [90] was followed for the MTT assay. The 3-(4,5-dimethylthiazol-2-yl)-2,5-diphenyl-2H-tetrazolium bromide (MTT) assay was used to demonstrate the anti-proliferative capabilities of TiO_2_ NPs against the MCF-7 breast cancer cell lines. MCF-7 cells were plated for this purpose in a 96-well plate with 100 μL of culture media at a density of 20,000 cells per well. The culture media was removed from the plate and replaced with fresh media containing various concentrations of the TiO_2_ NPs (12.5, 25, 50, 100 and 200 µg/mL), and cells were cultured for another 24 h at 37 °C in a 5% CO_2_ atmosphere. The adhering cells were then washed with phosphate buffer solution (PBS), followed by MTT stock solution (we removed the MTT reagent and then added 100 μL of solubilisation solution), after a period of time using gyratory shaker with gentle stirring to aid dissolving. Later, using an ELISA reader, absorbance was measured at the reference wavelengths of 570 nm.

#### 4.12.3. Morphological Study Using Fluorescence Microscopy

The ability of TiO_2_ NPs to induce apoptosis was assessed using the AO/EB double staining test (MCF-7 cells) by employing the method of [91]. Separated MCF-7 cells were planted in six-well plates and the corresponding IC_50_ doses of TiO_2_ NPs were added. The cells were then incubated for 24 h. Ethidium bromide (100 µg/mL) and acridine orange (100 µg/mL) fluorescent dye solutions were combined with the appropriate cell types. The cells were incubated for 30 min in the dark at 37 °C. A fluorescent microscope was afterwards used to view AO/EB staining.

#### 4.12.4. Cell Apoptosis Assay

The method described by [92] was followed for the cell apoptosis assay. Apoptotic cells were quantified by Annexin V-FITC and propidium iodide (PI) double staining, using an Annexin V-FITC apoptosis detection kit. MCF-7 Cells in a 6-well plate at a density of 0.5 × 106 cells/2 mL were incubated with an IC_50_ concentration of 120 µg/mL of TiO_2_ NPs for 24 h and then washed with PBS. An amount of 200 μL of trypsin-EDTA solution was added and they were incubated at 37 °C for 3–4 min. Then, we added 2 mL culture medium and harvested the cells directly into 12 × 75 mm polystyrene tubes. We centrifuged the tubes for five minutes at 300× *g* at 25 °C. We carefully decanted the supernatant. We washed the cells twice with PBS and decanted the PBS completely. We added 5 μL of FITC Annexin V and gently vortexed the cells and incubated them for 15 min at 25 °C in the dark. We added 5 μL of propium iodide (PI) and 400 μL of 1X binding buffer to each tube and vortexed gently. Then, we analysed them by flow cytometry immediately after the addition of PI.

## 5. Conclusions

The current study reports the green synthesised TiO_2_ nanoparticles using *Terenna asiatica* fruit extract. TiO_2_ NPs protected against NaNO_2_-induced oxidative stress in RBCs through their antioxidant potential. Most importantly, TiO_2_ NPs also showed anti-inflammatory and anticancer activities. Further understanding their mechanism of action is of great interest and would pave the way to validating their observed therapeutic potential.

## Data Availability

The data is available with Manjula M.V., Rajeshwar Achur and Devaraja S.

## Sample Availability

Samples of the compounds are available from the authors.
